# The Impact of Satellite Time Group Delay and Inter-Frequency Differential Code Bias Corrections on Multi-GNSS Combined Positioning

**DOI:** 10.3390/s17030602

**Published:** 2017-03-16

**Authors:** Yulong Ge, Feng Zhou, Baoqi Sun, Shengli Wang, Bo Shi

**Affiliations:** 1 National Time Service Center, Chinese Academy of Sciences, Xi’an 710600, China; geyulong15@mails.ucas.ac.cn; 2 Key Laboratory of Precise Navigation, Positioning and Timing Technology, Chinese Academy of Sciences, Xi’an 710600, China; 3 University of Chinese Academy of Sciences, Beijing 100049, China; 4 School of Information Science Technology, East China Normal University, 500 Dongchuan Road, Shanghai 200241, China; fzhou@gfz-potsdam.de; 5 German Research Centre for Geosciences GFZ, Telegrafenberg, Potsdam 14473, Germany; 6 Institute of Ocean Engineering, Shandong University of Science and Technology, Qingdao 266590, China; shlwang@sdust.edu.cn; 7 College of Geomatics, Shandong University of Science and Technology, Qingdao 266590, China; shibo@sdust.edu.cn

**Keywords:** GNSS, quad-constellation, time group delay (TGD), differential code bias (DCB), standard point positioning, precise point positioning

## Abstract

We present quad-constellation (namely, GPS, GLONASS, BeiDou and Galileo) time group delay (TGD) and differential code bias (DCB) correction models to fully exploit the code observations of all the four global navigation satellite systems (GNSSs) for navigation and positioning. The relationship between TGDs and DCBs for multi-GNSS is clearly figured out, and the equivalence of TGD and DCB correction models combining theory with practice is demonstrated. Meanwhile, the TGD/DCB correction models have been extended to various standard point positioning (SPP) and precise point positioning (PPP) scenarios in a multi-GNSS and multi-frequency context. To evaluate the effectiveness and practicability of broadcast TGDs in the navigation message and DCBs provided by the Multi-GNSS Experiment (MGEX), both single-frequency GNSS ionosphere-corrected SPP and dual-frequency GNSS ionosphere-free SPP/PPP tests are carried out with quad-constellation signals. Furthermore, the author investigates the influence of differential code biases on GNSS positioning estimates. The experiments show that multi-constellation combination SPP performs better after DCB/TGD correction, for example, for GPS-only b1-based SPP, the positioning accuracies can be improved by 25.0%, 30.6% and 26.7%, respectively, in the N, E, and U components, after the differential code biases correction, while GPS/GLONASS/BDS b1-based SPP can be improved by 16.1%, 26.1% and 9.9%. For GPS/BDS/Galileo the 3rd frequency based SPP, the positioning accuracies are improved by 2.0%, 2.0% and 0.4%, respectively, in the N, E, and U components, after Galileo satellites DCB correction. The accuracy of Galileo-only b1-based SPP are improved about 48.6%, 34.7% and 40.6% with DCB correction, respectively, in the N, E, and U components. The estimates of multi-constellation PPP are subject to different degrees of influence. For multi-constellation combination SPP, the accuracy of single-frequency is slightly better than that of dual-frequency combinations. Dual-frequency combinations are more sensitive to the differential code biases, especially for the 2nd and 3rd frequency combination, such as for GPS/BDS SPP, accuracy improvements of 60.9%, 26.5% and 58.8% in the three coordinate components is achieved after DCB parameters correction. For multi-constellation PPP, the convergence time can be reduced significantly with differential code biases correction. And the accuracy of positioning is slightly better with TGD/DCB correction.

## 1. Introduction

Currently, with more and more satellites joining the family of navigation systems, multiple global navigation satellite systems (multi-GNSS) standard point positioning (SPP) [1], precise point positioning (PPP) [2], precise orbit determination (POD) [3] and meteorology are becoming increasingly popular.

GNSS pseudorange observations are well known to exhibit systematic biases related to delays caused by internal electronic/hardware components of the overall signal generation, transmission and processing chain [4]. In the new multi-GNSS and multi-frequency context, observations from different constellations, signals, frequencies and channels need to be processed along with each other and a proper consideration of biases becomes mandatory for a consistent modeling of all observations. For all precise GNSS applications that are supported by code and carrier-phase observations, existing code biases represent a non-negligible error source. This includes ”time-oriented” applications such as high-precision GNSS satellite clock estimation [5] as well as time transfer among GNSS observing stations [6], but also code- and phase-based carrier phase ambiguities resolution. For ionosphere analysis, consideration of inter-frequency code biases has been an issue for a long time. For positioning applications, code biases should be considered [7].

GNSS satellite clock offsets in both broadcast and precise products are specific to the conventional signal or signal combination employed in their generation. It has been common practice to define GNSS clock offsets with respect to a dual-frequency ionosphere-free linear combination (LC) of conventional reference signals for this purpose, such as L1/L2 P(Y)-code for GPS, G1/G2 P-code for GLONASS, and E1/E5a or E1/E5b for Galileo. For Galileo, the precise clock products from the Multi-GNSS Experiment (MGEX) are based on the E1/E5a signal combination. For Galileo broadcast clock offsets, each Galileo satellite broadcasts its own clock correction data for all signals through the relevant signal, the F/NAV (Freely Accessible Navigation) and I/NAV (Integrity Navigation). F/NAV reports the clock parameters valid for the E1/E5a combination, and the I/NAV reports the parameters for the E1/E5b combination. Within the Galileo navigation message (RINEX format), the second parameter in the “Broadcast Orbit 5” record (“Data source”) indicates the frequency pair the stored satellite clock corrections are valid for [8]. Typically, we find the value of 258 corresponding to F/NAV and 513 or 517 corresponding to I/NAV. Unlike other GNSSs, BeiDou broadcast clock offsets are chosen in a different manner, which are referred to a single-frequency B3 signal [9,10]. However, similar to other GNSSs, the BeiDou clock offsets in precise products are referred to the B1/B2 dual-frequency ionosphere-free observations combination [11,12]. During the estimation process of satellite clock offsets, inter-frequency code biases are commonly ignored for reference signals mentioned before, which are assimilated into the satellite clock offsets. When using other signal or combined signals differing from the conventional reference signal or signal combination, the code biases, such as time group delay (TGD) or differential code bias (DCB), should be applied, which are essential for code-based positioning, time service and ionosphere modeling [13].

The broadcast TGDs, which are referenced to empirical absolute satellite biases, are commonly used to compensate code biases in real-time for single-frequency users. TGDs are initially provided by the control segment based on measurements made by the space vehicle (SV) contractor during SV manufacture. Although TGDs are calibrated before launch, there is always some variation that occurs once the satellites are in orbit [14,15]. GPS TGDs have been estimated and monitored by the Jet Propulsion Laboratory (JPL) for more than 15 years. Furthermore, more accurate code biases designated as DCBs are provided by GNSS communities to account for the same biases as TGDs, particularly for the post-processing applications [16]. DCBs are in a relative sense to reflect the differential code biases (satellites or receivers) between two different code observations obtained on the same or two different frequencies [17]. On 1 June 1998, International GNSS Service (IGS) started the ionosphere working group with the aim of estimating satellite DCBs and developing global ionosphere maps (GIM) based on GPS observations [18,19]. The GLONASS satellites DCBs have also been estimated by Center for Orbit Determination in Europe (CODE) since 2003 (see IGS Mail No. 4371). Within the Multi-GNSS Experiment (MGEX) [20] campaign launched by IGS [21], multi-GNSS DCBs have been derived from observations of the network. Details of the DCB estimation process are described in Montenbruck et al. [22]. The TGD/DCB corrections and their relationship to GPS have been analyzed and summarized in [10]. A correction model for BeiDou is given in Montenbruck and Steigenberger [9], it is only applicable to SPP. Guo et al. [10] extend the TGD and DCB correction models to various occasions for BeiDou positioning and first clearly figure out the relationship between TGDs and DCBs for BeiDou. However, such correction models are only applicable to BeiDou, not to other GNSSs, such as Galileo. Furthermore, the relationship between TGDs and DCBs for Galileo is not figured out, and the equivalence of TGD and DCB correction models for Galileo is not demonstrated in current literature. The TGD/DCB correction models and the effectiveness of broadcast TGDs and DCBs as well as their influences on single GNSS (e.g., Galileo) and multiple GNSSs (e.g., GPS + GLONASS + BeiDou + Galileo) positioning are presently not clear yet in current literature.

Within this contribution, we first provide a summary of the current available TGDs and DCBs (inter-frequency) for multi-GNSS, and describe their relationship combing theory with practice. Thereafter, the TGD and DCB correction models are developed to multi-GNSS code-based positioning scenarios. Furthermore, comprehensive analysis of the influence of code biases on multi-frequency combination SPP has been performed using quad-constellation GNSSs code observations as well as quad-constellation PPP.

## 2. Multi-GNSS TGDs/DCBs Correction Model

### 2.1. Methodology

To maintain consistent modeling of the pseudorange observations of all the four GNSSs, we use P1 and b1 (GPS L1 frequency: 1575.42 MHZ; GLONASS G1 frequency: 1602.00 MHZ; BDS B1 frequency: 1561.098 MHZ; Galileo E1 frequency: 1575.42 MHZ) to denote the pseudorange observation on the first frequency; P2 and b2 (GPS L2 frequency: 1227.60 MHZ; GLONASS G2 frequency: 1246.00 MHZ; BeiDou B2 frequency: 1027.14 MHZ; Galileo E5a frequency: 1176.45 MHZ) to denote the pseudorange observation on the second frequency; and P3 and b3 (BeiDou B3 frequency: 1268.52 MHZ; Galileo E5b frequency: 1027.14 MHZ) to denote the pseudorange observation on the third frequency.

In a multi-GNSS context, data processing poses the problem of mutual alignments of reference frames and time scales. Even though the broadcast orbits of GPS (WGS84), GLONASS (PZ90.11), Beidou (CGCS2000), and Galileo (GTRF) are formally referred to different reference frames, current realizations of these frames are very closely aligned with the International Terrestrial Reference Frame (ITRF), and they are commonly considered to agree at a few centimeter level [23], the difference in reference frames can hence be ignored in multi-GNSS SPP processing. For use within multi-GNSS, all broadcast and precise orbits should be aligned to a unique time scale, commonly referred to GPS time scale, which also forms the basis of all the observations. Computationally, the differences in time systems have been carried out by either solving for an additional receiver clock correction for each additional time system, or by solving for a receiver clock correction and the offsets to the other time systems. We take the former strategy in our data processing.

### 2.2. Undifferenced Pseudorange Observation Equations

Without loss of generality, the functional model of triple-frequency quad-constellation GNSS pseudorange observation *P_i_* (*i* = 1, 2, 3) can be expressed as:

(1) $$P_{1}=\rho +T+I_{1}+dt^{rcv}-dt^{sat}+B_{P_{1}}$$

(2) $$P_{2}=\rho +T+\alpha I_{1}+dt^{rcv}-dt^{sat}+B_{P_{2}}$$

(3) $$P_{3}=\rho +T+\beta I_{1}+dt^{rcv}-dt^{sat}+B_{P_{3}}$$

where indices *sat* and *rcv* refer to satellite and receiver, respectively; *ρ* is the true geometric distance between satellite and receiver; *T* is the slant troposphere delay; *I*_1_ is the first-order slant ionospheric delay on the first frequency; *α* and *β* are constant frequency-dependent multiplier factors (*α* = *f*_1_^2^/*f*_2_^2^, *β* = *f*_1_^2^/*f*_3_^2^, *k* = *f*_2_^2^/*f*_3_^2^); *dt^rcv^* and *dt^sat^* are the receiver and satellite clock offsets in meters, respectively; and $B_{P_{i}}$ (*i* = 1, 2, 3) is the code bias of both the receiver and satellite in meters. Code multipath and code noise are ignored in the above model for simplicity. Actually, the code bias of the receiver is the same for all of the common-view satellites with the same signals at each epoch, and they can be assimilated into the receiver clock offset without degrading the positioning vector in the positioning applications. Therefore, the code bias of the receiver is not considered any more, and $B_{P_{i}}$ (*i* = 1, 2, 3) stands only for the satellite part of the code bias in the following sections of this contribution.

### 2.3. TGD/DCB Correction Model for Multi-GNSS

TGD/DCB correction models for multi-GNSS are derived and extended for various occasions: correction models for either broadcast satellite clock or precise satellite clock users; correction models with either TGD or DCB parameters; and correction models for any single- and dual-frequency signals. In addition, the relationship between TGDs and DCBs for Galileo are explicitly figured out in this section. The formula can be simplified as:

(4) $$P_{1}=\rho +T+I_{1}+B_{P_{1}}$$

(5) $$P_{2}=\rho +T+\alpha I_{1}+B_{P_{2}}$$

(6) $$P_{3}=\rho +T+\beta I_{1}+B_{P_{3}}$$

(7) $$PC_{12}=\rho +T+B_{P_{12}}$$

(8) $$PC_{13}=\rho +T+B_{P_{13}}$$

(9) $$PC_{23}=\rho +T+B_{P_{23}}$$

where $PC_{ij}$ (*i*, *j* = 1, 2, 3) is the ionosphere-free code observable in meters. $B_{P_{ij}}$ (*i*, *j* = 1, 2, 3) is the code bias after ionosphere-free LC.

(1) GNSS TGD/DCB correction models for single- and dual-frequency users with broadcast satellite clock.

When GPS satellites are used, $B_{P_{i}}$ (*i =* 1, 2, 3) and $B_{P_{ij}}$ (*i*, *j* = 1, 2, 3) can be described as:

(10) $$B_{P_{1}}=-TGD\;\equiv \;\frac{1}{\alpha -1}DCB_{P_{1}P_{2}}$$

(11) $$B_{P_{2}}=-\alpha TGD\;\equiv \;\frac{\alpha }{\alpha -1}DCB_{P_{1}P_{2}}$$

(12) $$B_{P_{12}}=0$$

The relationship between TGD and DCB is $TGD=\frac{1}{1-\alpha }DCB_{P_{1}P_{2}}$. It is worth mentioning that P1-C1 bias corrections should be considered in some occasions. Detailed correction terms for various observations refer to Schaer [24].

When BDS satellites are used, $B_{P_{i}}$ (*i =* 1, 2, 3) and $B_{P_{ij}}$ (*i*, *j* = 1, 2, 3) can be described as:

(13) $$B_{P_{1}}=-TGD_{1}\;\equiv \;DCB_{P_{1}P_{3}}$$

(14) $$B_{P_{2}}=-TGD_{2}\;\equiv \;-DCB_{P_{2}P_{3}}$$

(15) $$B_{P_{3}}=0$$

(16) $$B_{P_{12}}=-\left[\frac{\alpha }{\alpha -1}TGD_{1}-\frac{1}{\alpha -1}TGD_{2}\right]\;\equiv \;-\left[\frac{\alpha }{\alpha -1}DCB_{P_{1}P_{3}}-\frac{1}{\alpha -1}DCB_{P_{2}P_{3}}\right]$$

(17) $$B_{P_{13}}=-\frac{\beta }{\beta -1}TGD_{1}\;\equiv \;-\frac{\beta }{\beta -1}DCB_{P_{1}P_{3}}$$

(18) $$B_{P_{23}}=-\frac{k}{k-1}TGD_{2}\;\equiv \;-\frac{k}{k-1}DCB_{P_{2}P_{3}}$$

The relationship between TGD and DCB are: $TGD_{1}=DCB_{P_{1}P_{3}}$, $TGD_{2}=DCB_{P_{2}P_{3}}$.

When Galileo satellites are used, $B_{P_{i}}$ (*i =* 1, 2, 3) and $B_{P_{ij}}$ (*i*, *j* = 1, 2, 3) can be described as:

(19) $$B_{P_{1}}=TGD_{2}\;\equiv \;\frac{1}{1-\beta }DCB_{P_{1}P_{3}}$$

(20) $$B_{P_{2}}=\alpha TGD{\;}_{1}\;\equiv \;\frac{1}{1-\alpha }\;DCB_{P_{1}P_{2}}$$

(21) $$B_{P_{3}}=\beta TGD_{2}\equiv \frac{\beta }{1-\beta }DCB_{P_{1}P_{3}}$$

According to the released Galileo ICD, the dual-frequency ionosphere-free pseudorange combinations (PCs) referring to E1/E5a and E1/E5b signals are free of code biases once the satellite clock offsets $dt_{IF(P_{1},P_{2}),brd}^{sat}$ and $dt_{IF(P_{1},P_{3}),brd}^{sat}$ are used. Hence, for dual-frequency users, the ionosphere-free PCs take the following derivations:

(22) $$B_{P_{12}}=0$$

(23) $$B_{P_{13}}=0$$

(24) $$B_{P_{23}}=-\left(\frac{\alpha k}{k-1}TGD_{1}-\frac{\beta }{k-1}TGD_{2}\right)\;\equiv \;-\frac{1}{k-1}\left(\frac{k}{\alpha -1}DCB_{P_{1}P_{2}}-\frac{\beta }{\beta -1}DCB_{P_{1}P_{3}}\right)$$

The relationship between TGD and DCB is $TGD_{1}=\frac{1}{\alpha \left(1-\alpha \right)}DCB_{P_{1}P_{2}}$, $TGD_{2}=\frac{1}{1-\beta }DCB_{P_{1}P_{3}}$, where *TGD*_1_ and *TGD*_2_ represent BGD(E1,E5a) and BGD(E1,E5b) from Galileo ICD, respectively.

(2) GNSS TGD/DCB correction models for single- and dual-frequency users with precise satellite clock. GPS TGD/DCB and GLONASS DCB correction models for single- and dual-frequency user with precise satellite clock is the same as Equations (10)–(12).

For BDS satellites,

(25) $$B_{P_{1}}=-\frac{1}{\alpha -1}\left(TGD_{1}-TGD_{2}\right)\;\equiv \;-\frac{1}{\alpha -1}DCB_{P_{1}P_{2}}$$

(26) $$B_{P_{2}}=-\frac{\alpha }{\alpha -1}\left(TGD_{1}-TGD_{2}\right)\;\equiv \;-\frac{\alpha }{\alpha -1}DCB_{P_{1}P_{2}}$$

(27) $$B_{P_{3}}=-\left(\frac{\alpha }{\alpha -1}TGD_{1}-\frac{1}{\alpha -1}TGD_{2}\right)\;\equiv \;-\left(\frac{\alpha }{\alpha -1}DCB_{P_{1}P_{3}}-\frac{1}{\alpha -1}DCB_{P_{2}P_{3}}\right)\;$$

(28) $$\begin{array}{l} B_{P_{13}}=\left(\frac{1}{\beta -1}-\frac{1}{a-1}\right)TGD_{1}+\frac{1}{\alpha -1}TGD_{2}\\ \;\equiv \;-\left(\frac{1}{\alpha -1}DCB_{P_{1}P_{2}}-\frac{1}{\beta -1}DCB_{P_{1}P_{3}}\right) \end{array}$$

(29) $$B_{P_{12}}=0$$

(30) $$\begin{array}{l} B_{P_{23}}=\frac{\alpha }{1-\alpha }TGD_{1}+\left(\frac{1}{k-1}+\frac{\alpha }{\alpha -1}\right)TGD_{2}\\ \;\equiv \;-\left(\frac{\alpha }{\alpha -1}DCB_{P_{1}P_{2}}-\frac{1}{k-1}DCB_{P_{2}P_{3}}\right) \end{array}$$

For Galileo satellites,

(31) $$B_{P_{1}}=\alpha TGD_{1}\;\equiv \;-\frac{1}{\alpha -1}DCB_{P_{1}P_{2}}$$

(32) $$B_{P_{2}}=\alpha^{2}TGD_{1}\;\equiv \;-\frac{\alpha }{\alpha -1}DCB_{P_{1}P_{2}}$$

(33) $$B_{P_{3}}=\left(\alpha TGD_{1}+\left(\beta -1\right)TGD_{2}\right)\equiv \;-\left(\frac{\alpha }{\alpha -1}DCB_{P_{1}P_{3}}-\frac{1}{\alpha -1}DCB_{P_{2}P_{3}}\right)\;$$

(34) $$B_{P_{12}}=0$$

(35) $$B_{P_{13}}=\alpha TGD_{1}\;-TGD_{2}\;\equiv \;-\left(\frac{1}{\alpha -1}DCB_{P_{1}P_{2}}-\frac{1}{\beta -1}DCB_{P_{1}P_{3}}\right)$$

(36) $$B_{P_{23}}=\left(\alpha^{2}TGD_{1}-\frac{\beta -1}{k-1}TGD_{2}\right)\;\equiv \;-\left(\frac{\alpha }{\alpha -1}DCB_{P_{1}P_{2}}-\frac{1}{k-1}DCB_{P_{2}P_{3}}\right)$$

In general, it is common to use the TGDs parameters from the RINEX navigation message for GPS, BDS and Galileo SPP, the real-time positioning application, etc. because DCBs products cannot be obtained in real-time. For PPP and the post positioning processing applications, using DCBs products is the best choice. The DCBs are systematically biased from the TGDs with constant offsets [10]. The positioning applications will not be affected due to the common code biased of the constellation will be absorbed by receiver clock errors, while precise timing will be affected. It should be mentioned that the DCBs are systematically biased from the TGDs with constant offsets, such as TGD1 and TGD2 of BDS will not be equal to $DCB_{P_{1}P_{3}}$ and $DCB_{P_{2}P_{3}}$, respectively. However, it does not matter to the positioning applications due to common biases of the constellation will absolutely be absorbed by receiver clock errors.

## 3. Data and Processing Strategy

All observation data sets used in this study were collected by various organizations contributing to MGEX stations which was set-up by the IGS in 2011 to track, collect, and analyze all available GNSS signals [20]. The MGEX network has grown to more than 110 stations now supporting at least one of the new navigation systems (BeiDou, Galileo and QZSS) in addition to the legacy GPS, GLONASS and SBAS (Satellite Based Augmentation Systems). The quad-constellation satellite orbits and clock offsets are corrected by the broadcast ephemris provided by MGEX (ftp://cddis.gsfc.nasa.gov/pub/gps/data/campaign/mgex/daily/rinex3/2015/brdm/), or the precise orbit and clock products at intervals of 15 min and 30 s, respectively, provided by The German Research Center for Geosciences (GFZ). The detailed observation model and data processing strategies are summarized in Table 1 which is for the positioning at the user end. Notably, GLONASS does not require relativistic clock correction on the broadcast satellite clock offset at the user end. GPS, BDS and Galileo TGD parameters can be obtained in brdmddd0.yyp file, where *ddd* and *yy* indicate day of year (DOY) and the two-digit year. Currently, both GNSS satellites and receiver biases from weekly averages of daily DCBs are provided at ftp://cddis.gsfc.nasa.gov/pub/gps/products/mgex/dcb/. GNSS DCBs was extracted from the annual file “MGEX2015_all.bsx.Z” in this study.

Most of the GNSS observation errors (troposphere delay, ionosphere delay, multipath effect, etc.) have something to do with the elevation angle of satellites. In order to weaken these errors, stochastic models based on the elevation angle of satellite can be established. Elevation-angle based stochastic models mainly include trigonometric function model and exponential function model. In this study, we use the sine function based elevation-angle stochastic model:

(37) $$\sigma^{2}=\frac{\sigma_{0}^{2}}{\sin^{2}\theta }$$

where *θ* is the elevation angle of the satellite, and $\sigma_{0}^{2}$ is the prior variance of observations.

Generally, the multipath and the large observation noise usually exist at low elevation angle. In order to reduce the weight of the observation with lower elevation angle, we define the weight segmentation. The corresponding code and carrier phase variance matrix are:

(38) $$\sigma^{2}=\left\{ \begin{array}{l} \begin{array}{cc} \frac{\sigma_{0}^{2}}{\sin\theta } & \;\theta >\alpha \end{array}\\ \begin{array}{cc} \frac{\sigma_{0}^{2}}{\sin^{2}\theta } & \theta <\alpha \end{array} \end{array}\right.,$$

*α* is the elevation angle threshold, it is set to be 30° generally. When adopting the pseudorange and carrier phase observation at the same time, the variance-covariance expression is:

(39) $$\sigma_{i}^{2}=\left[\begin{array}{cc} \sigma_{P,i}^{2} & 0\\ 0 & \sigma_{\phi ,i}^{2} \end{array}\right]$$

where $\sigma_{P,i}^{2}$, $\sigma_{\phi ,i}^{2}$ are the a priori variances of code and carrier phase observations, respectively.

It is worth mentioning that different GNSS system have different observation a priori variances. For the GPS and GLONASS code and carrier phase observation, the precision is set to be 0.3 m and 0.002 m, respectively. Since the BDS satellite orbit and clock are at a relatively lower accuracy, its measurements are down-weighted. That is, the phase observation precision is set to be 0.004 m and the code observation precision is set to be 0.6 m for BDS and Galileo [25].

**Figure 1:**
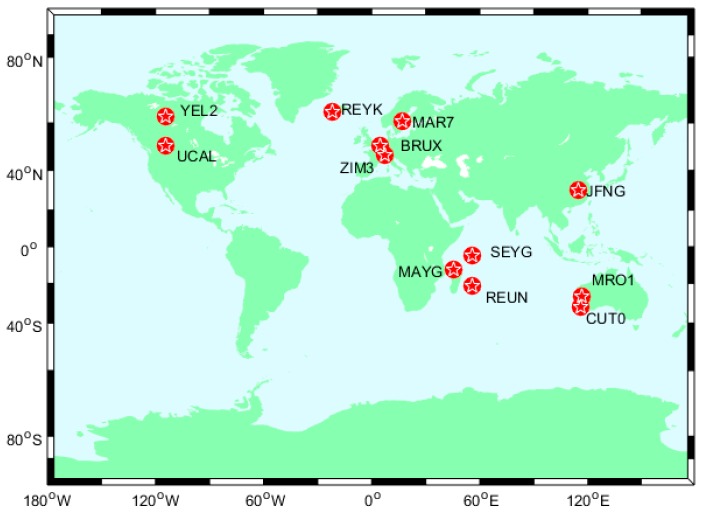
The distribution of stations from MGEX.

The BeiDou and Galileo antenna offsets recommended by the MGEX project are used to correct the PCOs of BeiDou and Galileo satellites [31]. The distribution of eight stations from MGEX are shown on Figure 1.

To investigate the influence of code bias on different constellation combinations positioning, three different schemes [10], pseudorange without TGDs or DCBs corrections, TGD corrected and DCB corrected, are described in Table 2. G, R, C and E represent GPS, GLONASS, BDS and Galileo, respectively.

## 4. Experimental Results and Analysis

### 4.1. Performance of SPP with Broadcast Ephemeris

#### 4.1.1. Single-Frequency

**Figure 2:**
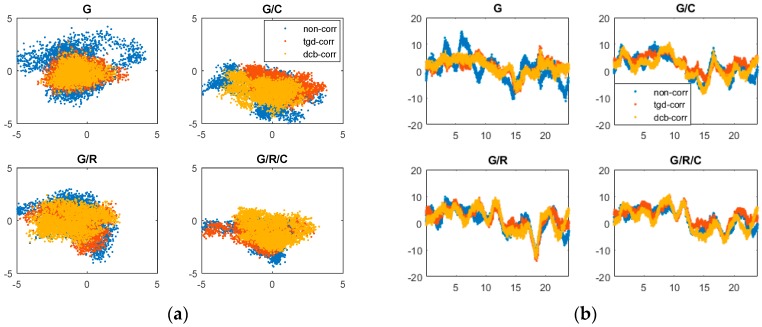
Horizontal (**a**); and vertical (**b**) positioning error scatters of b1 SPP with different schemes in four different constellation combinations. (**a**) For each plot, the horizontal and vertical axes represent, respectively, the N and E component error (unit: m). (**b**) For each plot, the horizontal and vertical axes represent, respectively, the universal time (unit: h) and the positioning error of up component error (unit: m).

**Figure 3:**
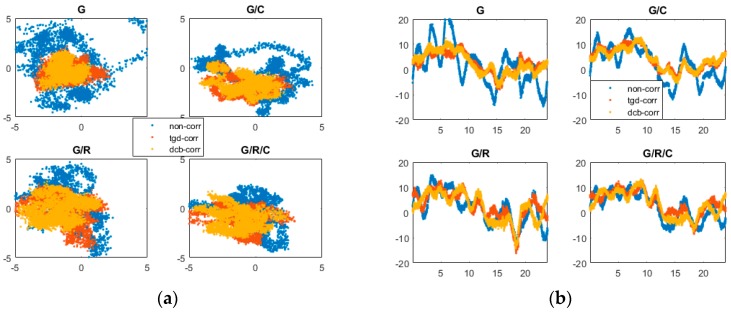
Horizontal (**a**); and vertical (**b**) positioning error scatters of b2 SPP with different schemes in four different constellation combinations. (**a**) For each plot, the horizontal and vertical axes represent, respectively, the N and E component error (unit: m). (**b**) For each plot, the horizontal and vertical axes represent, respectively, the universal time (unit: h) and the positioning error of up component error (unit: m).

Figure 2 and Figure 3 show the positioning errors of one particular day (2 May 2015) for the north (N), east (E), and up (U) component of b1 and b2 based different constellation combinations SPP at CUT0 station. Different constellation combinations SPP results with b1 and b2 on other stations show similar features, thus would not be presented herein. The root mean square (RMS) errors of the single-frequency SPP are calculated, and the mean values of all tests in different constellation combinations are summarized in Table 3. As shown in Figure 2 and Figure 3 and Table 3, the horizontal positioning error can reach meter-level, while the vertical positioning error is relatively large. For b1 and b2 different constellation combination SPP, solution of “non-corr”, the values of RMS can reach 2–3 m in horizontal, and 5–10 m in vertical. It is obvious that the positioning accuracy benefits from multi-GNSS combinations. Significant improvements can be seen in the “tgd-corr” and “dcb-corr” where the code biases are corrected with TGD and DCB parameters. The values of RMS can reach 1–2 m in horizontal and about 5 m in vertical after TGD/DCB correction. For GPS-only b1-based SPP, the positioning accuracy can be improved by 25.0%, 30.6% and 26.7%, respectively, in the N, E, and U components, after the differential code biases correction. For GPS/GLONASS/BDS b1-based SPP, the positioning accuracy can be improved by 16.1%, 26.1%, and 9.9%, respectively, in the N, E, and U components.

As shown in Table 3, the positioning accuracy of multi-GNSS combination SPP performance benefits from TGD/DCB correction. For b3-based SPP, GPS/BDS/Galileo, GPS/BDS and Galileo SPP were tested, this is because that GLONASS has no triple-frequency signal. The result of GPS/BDS b3-based SPP is mainly affected by BDS satellites for that there are few triple-frequency GPS satellites and the TGD/DCB was not corrected for GPS b3 pseudorange in the test. Hence the positioning results are unaffected by the differential code bias since the broadcast satellite clock corrections referring to B3 pseudorange.

On the other hand, to investigate the impact of differential code bias on Galileo satellites positioning for single frequency user, GPS/BDS/Galileo SPP and GPS/BDS/GLONASS/Galileo SPP were tested due to fewer Galileo satellites. As we can see from Table 3, compared with GPS/BDS b1-based SPP, the positioning accuracy of GPS/BDS/Galileo b1-based SPP was improved not significant in the first schemes. For GPS/BDS/Galileo b3-based SPP, the positioning accuracy can be improved by 0.2%, 1.1% and 0.1%, respectively, in the N, E, and U components, after TGD correction. It should be mentioned that the positioning results of GPS/BDS b3-based SPP are unaffected by the differential code bias. The reason is the same as the previous one. The performance improvement of GPS/BDS/Galileo b3-based SPP after differential code bias correction are mainly affected by Galileo satellites differential code bias correction. The positioning accuracy can be improved by 2.0%, 2.0% and 0.4%, respectively, in the N, E, and U components, after DCB correction.

**Figure 4:**
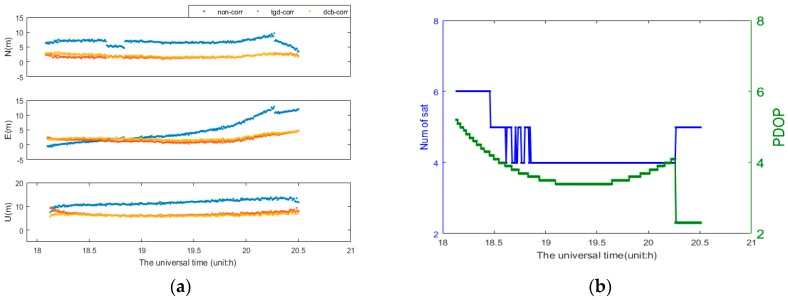
Galileo-only b1-based SPP positioning errors of BRUX for three different processing cases (**a**); and Satellite number and PDOP at BRUX (**b**).

For Galileo-only SPP, the daily solution cannot be presented due to few Galileo satellites. Taking the dateset from MGEX station BRUX during time 18:06–20:30 on DOY 184, 2016 as an example, the Galileo satellite number and PDOP are displayed in Figure 4b. The PDOP values vary between 2.2 and 5.1. The Galileo-only b1-based positioning errors for three different processing cases are shown in Figure 4a. The positioning accuracy can be improved significantly with TGD/DCB correction. The mean RMS values of Galileo-only positioning are presented in Table 3. Comparing with the first scheme (“non-corr”), the accuracy of b1-based SPP are improved about 46.9%, 34.1% and 34.9% with TGD correction, as well as 48.6%, 34.7% and 40.6% with DCB correction, in the N, E, and U components, respectively. The accuracy of Galileo-only b2- or b3-based positioning are subject to different degrees of influence with TGD/DCB corrections.

#### 4.1.2. Dual-Frequency

**Figure 5:**
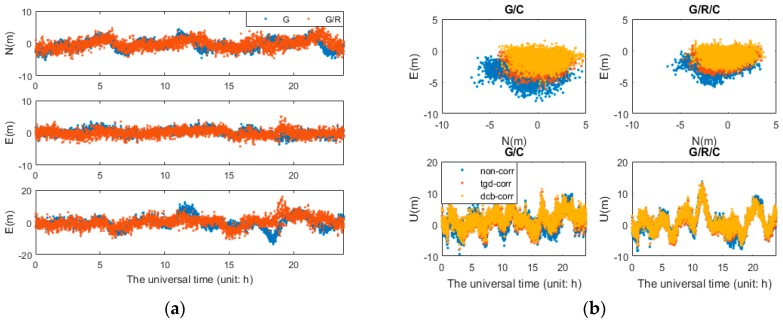
Positioning error series of b1b2 SPP in multi- constellation combinations: (**a**) description of GPS and GPS/GLONASS combinations; and (**b**) description of GPS/BDS and GPS/GLONASS/BDS combinations.

**Figure 6:**
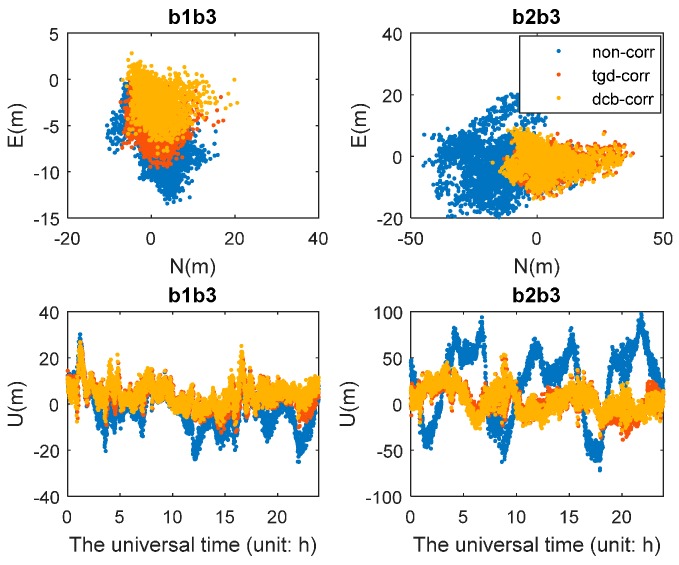
Positioning error scatters of b1b3 and b2b3 SPP with different schemes in G/C constellation combinations. For each plot, the upper and lower plots represent, respectively, horizontal positioning error scatters and Vertical positioning error scatters.

As can be seen in Figure 5, the positioning error of b1b2 ionosphere-free combined SPP in different constellation combinations at CUT0 station are presented. Figure 6 shows the position error of b1b3 and b2b3 ionosphere-free combined SPP in different constellation combinations. The mean values (RMS error) of all dual-frequency SPP tests in different constellation combinations are given in Table 4. For Figure 5, the positioning results of b1b2 ionosphere-free combined SPP in GPS and GPS/GLONASS combinations are unaffected by the different code bias since the broadcast satellite clock corrections refer to dual-frequency ionosphere-free LC. As shown in Table 4, the b1b2-based SPP shows better performance, while that of b1b3- and b2b3-based SPP show poor performance relatively, especially that of b2b3-based SPP. This is because that the observation noise is amplified. Compared with the first schemes, the positioning accuracy performance is better after TGD/DCB correction, especially the third schemes. For example, for b1b2-based GPS/BDS SPP, the positioning accuracy can be improved by 13.5%, 25.3%, and 3.8%, and 15.1%, 37.1%, and 5.3% in N, E, and U components after TGD and DCB correction, respectively. This may be attributed to the more accurate DCB products provided by MGEX. Compared with the results of b1b2-based ionosphere-free LC SPP, the performance of single-frequency SPP is slightly poor for the poor accuracy of Klobuchar model. For b1b3-based GPS/BDS SPP, the positioning accuracy can be improved by 33.9%, 20.4% and 29.3%, respectively, in N, E, and U components after TGD correction, and be improved by 36.9%, 47.3% and 43.2% respectively, in N, E, and U components after DCB correction. For b2b3-based GPS/BDS SPP, the positioning accuracy can be improved by 60.9%, 26.5% and 58.8%, respectively, in N, E, and U components after TGD correction, and be improved by 71.8%, 62.32% and 81.45%, respectively, in N, E, and U components after DCB correction. It should be pointed out that, since the large amplification factor of b2b3 combination, the b2b3-based SPP is much more sensitive to the code biases. In general, the positioning accuracy of multi-GNSS dual-frequency combination SPP is slightly worse than single-frequency SPP. However, the positioning accuracy of triple-constellation b1b2-based SPP can reach 1–2 m in horizontal and 2–3 m in vertical. The positioning accuracy of GPS/BDS b1b3-based SPP can reach 2–4 m in horizontal and 5–6 m in vertical and the b2b3-based SPP can reach 5–6 m in horizontal and 6–7 m in vertical after TGD/DCB correction. It should be noted that the results of GPS/BDS b1b2 combination are slightly poorer without TGD/DCB correction than GPS. It can also explain the importance of TGD/DCB correction for BDS positioning.

As we can see in Table 4, compared with GPS/BDS b1b2- and b1b3-based SPP, the RMS for the three-dimension (3-D) position of GPS/BDS/Galileo b1b2- and b1b3-based SPP are improved by 6.2% and 3.3%, respectively. It is worth noting that Galileo b1b2- and b1b3-based SPP are unaffected by differential code bias correction according to Equations (22) and (23). For b2b3-based SPP, the GPS/BDS/Galileo combination SPP slightly improves the 3-D positioning accuracy over the GPS/BDS combination for more than 7.8% in the third schemes (DCB correction).

### 4.2. Performances of PPP

**Figure 7:**
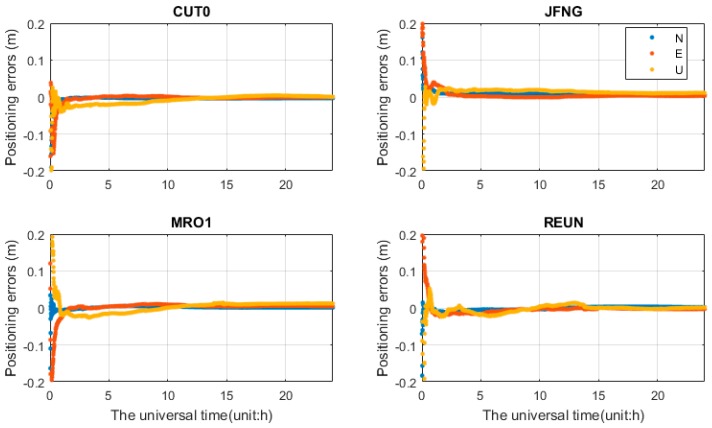
Positioning error (N, E, and U) of b1b2 quad-constellation PPP. For each plot, the horizontal axis represents the universal time (unit: h), and the vertical axis represents the corresponding positioning error (unit: m).

**Figure 8:**
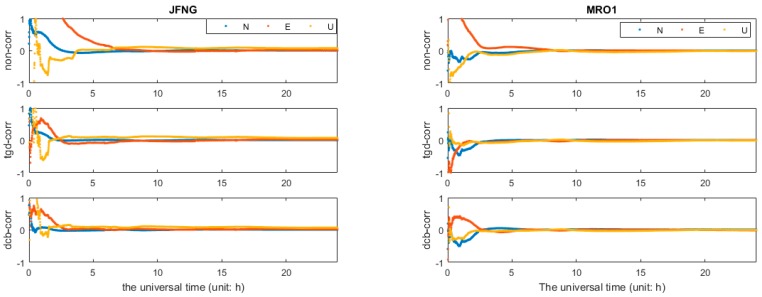
Positioning results (N, E, and U) of b1b3 GPS/BDS/Galileo PPP (unit: m).

**Figure 9:**
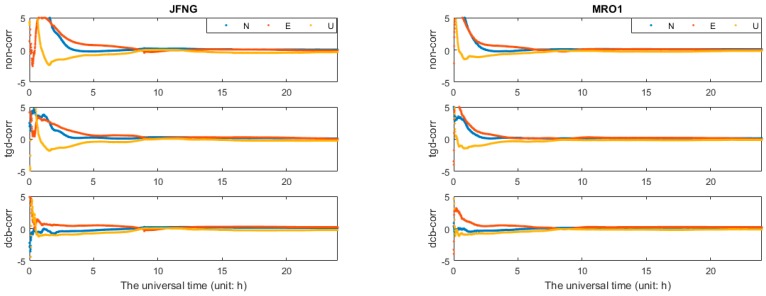
Positioning results (N, E, and U) of b2b3 GPS/BDS/Galileo PPP (unit: m).

Figure 7, Figure 8 and Figure 9 show the positioning solution of b1b2-, b1b3-, and b2b3-based PPP in multi-constellation. The RMS errors of dual-frequency PPP in multi-constellation combinations are calculated, and the mean values of all PPP tests after convergence are summarized in Table 5. The results were statistically calculated from one hour after positioning. As show in Table 5, b1b2-based PPP show the best performance. The positioning accuracy reaches about 0.5–2 cm in horizontal and 2–5 cm in vertical. The positioning accuracy of multi-constellation PPP shows better performance than single constellation PPP. Compared with single constellation PPP, Multi-constellation PPP can obviously improve the convergence time [32]. The BDS-only PPP has poorer accuracy than the GPS-only PPP. This is most probably because the poor orbit accuracy of GEO, smaller number of satellites, especially outside East-Asian/Australian region and presently, the lack of precise PCO and PCV corrections are now available for BDS satellites and receiver [25]. The results of b1b2-based PPP are unaffected by differential code bias. This is because that the precise satellite clock corrections refer to b1b2 ionosphere-free LC.

**Figure 10:**
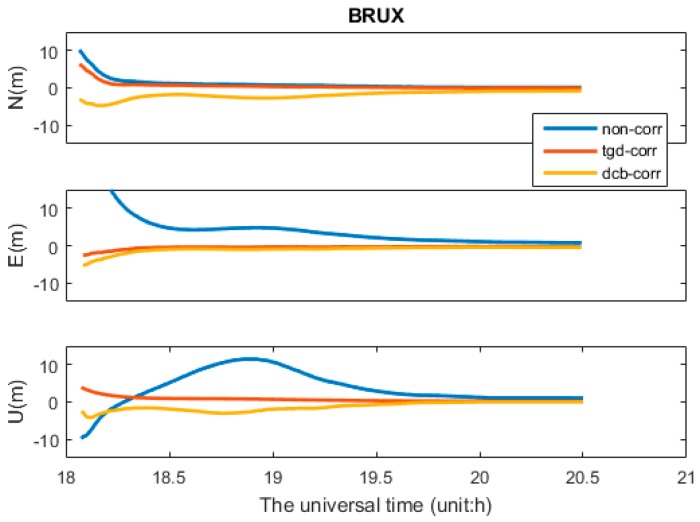
Positioning results (N, E, and U) of b1b3-based Galileo PPP.

For b1b3- and b2b3-based PPP, as shown in Figure 8 and Table 5, the positioning accuracy is significantly worse than b1b2-based PPP, while b2b3-based PPP shows the worst performance. It can be seen that b1b3-based PPP takes much longer time to converge in the first schemes (“non-corr”). The convergence time were improved obviously after TGD/DCB parameters correction. The results of b1b3-based PPP need five hours or longer to reach the centimeter level without differential code bias, while it takes only about 2 h after TGD/DCB correction. Compared with BDS-only PPP, GPS/BDS PPP shows better performance. The triple-constellation (GPS/BDS/Galileo) PPP further increases the positioning accuracy over the dual-constellation (GPS/BDS) PPP. The result of triple-constellation PPP after TGD/DCB correction show better performance than the result of triple-constellation PPP without differential code biases correction. Figure 10 indicates the positioning results of Galileo-only b1b3-based PPP. Combined with Figure 4b, the positioning accuracy is poor due to few Galileo satellites. The difference of b1b3-based PPP solutions between the first scheme (“non-corr”) and TGD/DCB correction reaches a few meters at first few hours. After a few hours smoothing, the differences are decreased to few millimeters. The convergence time are reduced significantly with TGD/DCB corrections. The same feature can be observed in the b2b3 PPP with slightly larger differences, and will not be presented herein.

**Figure 11:**
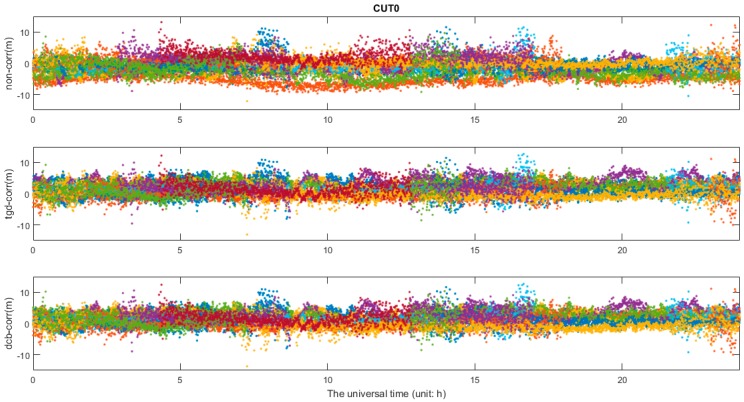
Pseudorange residuals: b1b3 GPS/BDS/ Galileo PPP solutions on CUT0 station.

**Figure 12:**
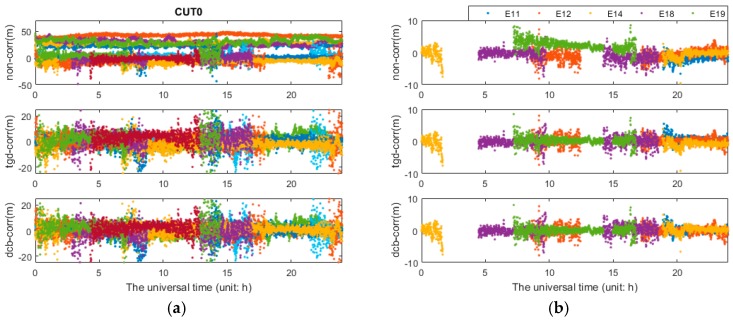
Pseudorange residuals of PPP solutions on CUT0 station: (**a**) description of b2b3 GPS/BDS/Galileo PPP solutions; and (**b**) description of b1b3 GPS/BDS/Galileo PPP solutions.

The differential code bias would affect positioning results; in addition, it can be absorbed by other parameters, such as receiver clock, ambiguity and tropospheric delay or be reflected in the residual. Figure 11 shows the pseudorange residuals of b1b3 PPP in GPS/BDS/Galileo combination. As can be seen in Figure 12, the pseudorange residuals of b2b3 PPP in GPS/BDS/Galileo combination and Galileo satellites at b1b3 PPP solution are presented, the same feature can be observed in b2b3 PPP with slightly larger differences, which will not be presented herein. As we can see in Figure 11 and Figure 12, the residuals of the first schemes (“non-corr”) show the worst performance, especially for the pseudorange residuals of b2b3-based PPP. It is obvious that there are some systematic biases. The pseudorange residuals fluctuate around zero after TGD/DCB parameters correction, and the residuals values are significantly reduced. The Galileo satellites in the relatively small number of satellites can be observed in each session, and even unobserved. As part of the Galileo navigation satellites, ephemeris has not been provided; the residuals of some Galileo satellites are not affected by the TGD correction. The residual of Galileo shows the same feature with other satellites. On the other hand, the residuals of the first schemes are larger, which demonstrates that the initial positioning accuracy has been greatly affected. Thus, the convergence time is slow. In other words, the convergence time and the accuracy of positioning could be effectively reduced using TGD/DCB parameters.

The unmodeled differential code bias can be absorbed into receiver clock bias as well as the float ambiguities [10]. Thus, we should be very careful to take this issue into account when multi-GNSS is used for high precision timing applications. Compared with the result of the differential code biases uncorrected, the differences of the clock range up to 10–20 ns with the differential code biases corrected by DCB parameters for all tests. The tropospheric differences between the first schemes and the other two schemes, the values of difference are a few millimeters for all tests. The second and third schemes show the same feature during the whole period.

### 4.3. Kinematic Results and Analysis

**Figure 13:**
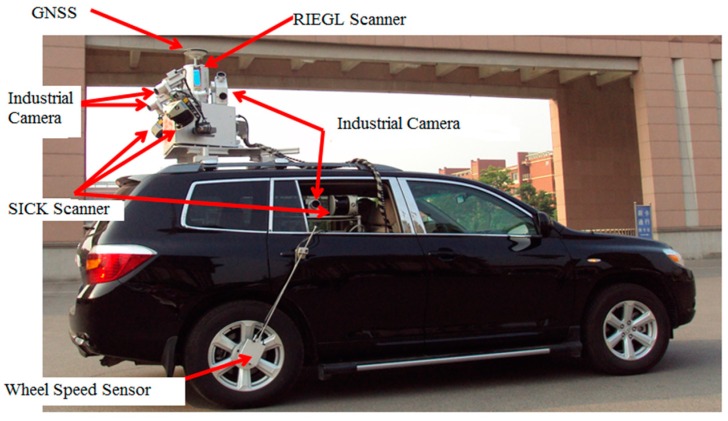
V-Surs I vehicle-borne three-dimensional mobile surveying system.

**Figure 14:**
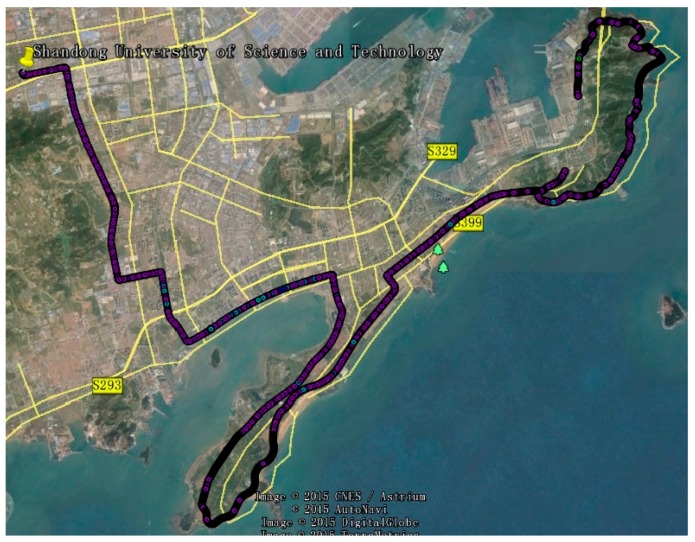
The trajectory of this experiment.

The GNSS data were collected from the V-Surs I vehicle-borne three-dimensional mobile surveying system for about 3 h while the vehicle was moving. As show in Figure 13, this system has been researched and developed by Shandong University of Science and Technology and the company of Supersurs mobile surveying service, it is equipped with a three-system receiver of NovAtel Propak6 and inertial measurement unit (IMU) of span LCI type. The type of antenna and the sampling rate of the receiver are NOV703GGG and 1 s, respectively. The trajectory of this experiment is shown in Figure 14.

**Figure 15:**
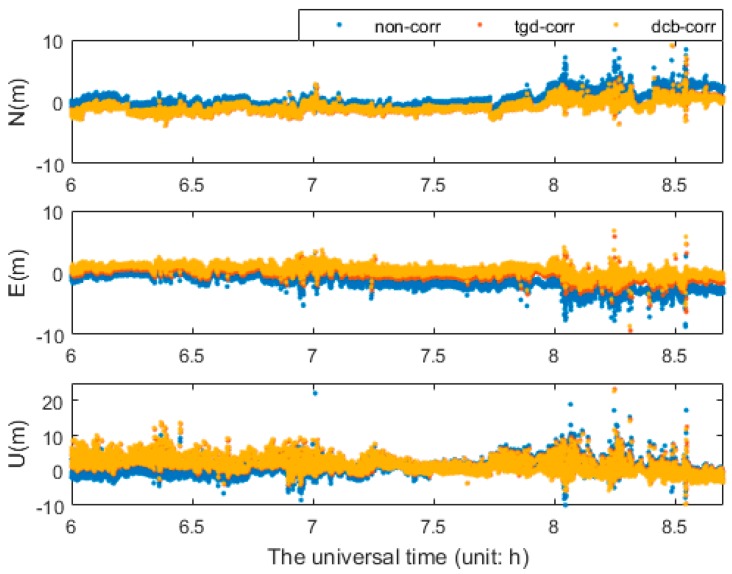
Positioning error series of b1b2 kinematic GPS/BDS/GLONASS SPP.

To investigate the impact of differential code biases on kinematic positioning, a triple-constellation SPP test was conducted at Qingdao, China on 30 April 2015. We used GNSS/INS tightly coupled resolution of Inertial Explorer 8.60 software (IE 8.60) to resolve these data, and the results were regarded as the external reference values. The positioning errors of GPS/BDS/GLONASS SPP show better performance after TGD/DCB correction in Figure 15. The RMS kinematic values using the positioning errors in 3 h are presented in Table 6. The results of uncorrected code biases seriously degrade the positioning accuracy. With the inclusion of the positioning accuracy with DCB/TGD correction, the triple-constellation b1b2-based SPP with DCB parameters correction improves the positioning accuracies about 63.8%, 72.7%, and 10.3%, respectively, in the three coordinate components, and the positioning accuracy can be improved by 65.1%, 72.1%, and 8.8% in N, E, and U, respectively, with TGD parameters correction. Test using b1 and b2 combination on multi-GNSS combination shows similar feature and will not be presented herein.

## 5. Summary

This paper introduces research regarding the status of multi-constellation (GPS + BDS + GLONASS + Galileo) timing group delay (TGD) and differential code bias (DCB) parameters, and then reveals the relationship between TGDs and DCBs for Galileo. Multi-GNSS TGD/DCB correction models for any single- (b1, b2, and b3) and dual-frequency (b1b2, b1b3, and b2b3) combinations from triple-frequency GNSS signals are assessed by three different schemes, in which the differential code biases are either ignored (“non-corr”), or corrected with TGD (“TGD-corr”) or DCB (“DCB-corr”) parameters. The model is extended to SPP/PPP processing with observations from single-, dual-, triple- or quad-constellations. Static datasets collected at eight stations over thirty consecutive days as well as a kinematic experimental dataset are used to fully evaluate the influence of positioning accuracy with TGD/DCB correction.

Comparative analysis of the influence of differential code biases on multi-GNSS combination (GPS, BDS, GPS + BDS, GPS + GLONASS, GPS + BDS + GLONASS, GPS + BDS + Galileo, and GPS + BDS + GLONASS + Galileo) positioning accuracy reveals that, for SPP with broadcast or precise orbit and clock, the positioning accuracy of GPS-only single frequency SPP can reach 2–3 m in horizontal and 5–10 m in vertical without the differential code biases, while the positioning accuracy can reach 1–2 m in horizontal and 5 m in vertical after TGD/DCB correction. The accuracy of Galileo-only b1-based SPP are improved about 48.6%, 34.7% and 40.6% with DCB correction, respectively, in the N, E, and U components. Multi-GNSS combination SPP achieves obviously better positioning accuracy than GSP-only SPP at three different schemes. For example, compared with b1-based GPS-only SPP, the accuracy of the b1-based GPS/BDS/GLONASS combination SPP can be improved by 23.5%, 8.0%, 17.5% in the three coordinate components, respectively. The uncorrected code biases seriously degrade the positioning accuracy of multi-GNSS combination dual-frequency SPP, especially for the b2b3-based SPP. For example, the positioning accuracy of GSP/BDS b2b3-based can be improved by 71.8%, 62.32% and 81.45%, respectively, in the three coordinate components. It is noted that the accuracy of static positioning after adding Galileo are not significant due to that there are fewer Galileo satellites currently in orbits. For GPS/BDS/Galileo b3-based SPP, the positioning accuracy can be improved by 2.0%, 2.0% and 0.4%, respectively, in the N, E, and U components, after Galileo satellites DCB correction. Compared to GPS/BDS b2b3-based SPP, GPS/BDS/Galileo b2b3-based SPP improves the 3-D positioning accuracy by 7.6% in the third schemes. The multi-GNSS ionosphere-free LC PPP positioning results indicate that the differential code bias has no influence on b1b2-based PPP, the positioning accuracy of b1b3- or b2b3-based PPP while the convergence time has been greatly improved by the TGD/DCB correction. The differences in PPP coordinate solutions are very small after convergence. The effect of coordinates parameters is mainly reflected in the initialization phase. It is interesting to note that the differential code biases do not matter to the positioning applications since the biases will be partly absorbed by other parameters such as receiver clock bias, tropospheric delay and carrier phase ambiguities. For kinematic positioning, GPS/GLONASS/BDS combination b1b2-based SPP with DCB parameters correction improves the positioning accuracy about 63.8%, 72.7%, and 10.3%, respectively, in the three coordinate components, and the positioning accuracy can be improved by 65.1%, 72.1%, and 8.8% in N, E, and U, respectively, with TGD parameters correction.

In general, for both the static and kinematic positioning, the performances have been improved significantly after TGD/DCB correction. It is unwise to ignore the differential code biases in the applications of multi-GNSS positioning, precise timing and tropospheric delay estimation.

## Figures and Tables

**Table 1:** Summary of observation model and data processing strategies for SPP and PPP.

Item	Descriptions
Number of stations	12
Date span	1–30 May 2015; 1–30 July 2016
Signal selection	GPS: L1/L2/L5; GLONASS: L1/L2; BeiDou: B1/B2/B3; Galileo: E1/E5a/E5b
Sampling interval	30 s
Elevation cut off	10°
Time system	GPS time
Tropospheric delay	Dry component: corrected with GPT model [26], wet component: estimated as random-walk process, GMF mapping function applied.
First order ionospheric delay	Single-frequency SPP:
	GPS/GLONASS: Klobuchar model
	BDS: Klobuchar model [27]
	Galileo: The NeQuick model [28]
	Dual-frequency: First order eliminated by ionosphere-free combination
Relativistic effect	IERS2010 [29]
Sagnac effect	IERS2010 [29]
Phase wind-up effect	Corrected [30]
Satellite PCO and PCV	GPS and GLONASS: Fixed to igs08_1861.atx values;
Tide displacement	IERS2010 [29]
Station reference coordinates	IGS SINEX solutions or daily GPS-only PPP solutions

**Table 2:** Summaries of the processing strategy.

Different Constellation Combinations	Proc. Mode	Combination	Schemes	Comments
G, C, E,	SPP	Single-freq: b1, b2, b3;	non-corr	non-corr: pseudorange without TGDs or DCBs corrections
G/R,				
G/C,		dual-freq: b1b2, b1b3, b2b3	tgd-corr	tgd-corr: TGD corrected
G/R/C,	PPP			
G/C/E,		dual-freq: b1b2, b1b3, b2b3	dcb-corr	dcb-corr: DCB corrected
G/R/C/E				

**Table 3:** RMS of single-frequency standard point positioning (unit: m).

Combinations	Scheme	b1			b2			b3		
			N	E	U	N	E	U	N	E	U
G	non-corr	2.712	1.830	7.170	4.455	2.937	11.477	-	-	-
G	tgd-corr	1.912	1.319	5.456	2.842	1.284	8.091	-	-	-
G	dcb-corr	2.034	1.269	5.254	2.676	1.363	6.186	-	-	-
E	non-corr	10.00	7.60	16.74	9.61	8.84	18.05	9.67	8.84	17.93
E	tgd-corr	5.41	4.03	11.03	6.25	4.75	13.36	5.68	4.37	11.84
E	dcb-corr	5.12	3.90	10.92	5.76	4.53	12.77	5.49	4.27	11.95
G/C	non-corr	2.125	1.805	6.251	3.313	2.403	9.843	4.044	3.836	11.458
G/C	tgd-corr	1.891	1.286	5.639	2.761	1.483	8.385	-	-	-
G/C	dcb-corr	1.791	1.312	5.491	2.528	1.528	6.437	-	-	-
G/R	non-corr	2.413	1.710	6.307	3.293	1.729	8.581	-	-	-
G/R	tgd-corr	2.250	1.349	5.838	3.293	1.729	8.581	-	-	-
G/R	dcb-corr	2.112	1.202	5.299	2.759	1.381	6.352	-	-	-
G/R/C	non-corr	2.074	1.665	5.915	3.033	2.179	9.038	-	-	-
G/R/C	tgd-corr	2.062	1.382	5.678	3.032	1.694	8.432	-	-	-
G/R/C	dcb-corr	1.740	1.230	5.327	2.588	1.442	6.335	-	-	-
G/C/E	non-corr	2.150	1.741	6.040	3.441	2.570	7.212	3.910	3.711	10.956
G/C/E	tgd-corr	1.759	0.973	5.347	2.412	1.193	6.193	3.899	3.670	10.944
G/C/E	dcb-corr	1.630	0.814	5.189	2.294	1.035	6.225	3.830	3.634	10.912
G/R/C/E	non-corr	1.885	1.367	5.493	2.977	2.106	6.726			
G/R/C/E	tgd-corr	1.755	0.990	5.335	2.396	1.175	6.160			
G/R/C/E	dcb-corr	1.610	0.801	5.177	2.246	0.913	6.120			

“-” represents no corresponding combination of results, the same below.

**Table 4:** RMS of dual-frequency standard point positioning (unit: m).

Combinations	Scheme	b1b2			b1b3			b2b3		
		N	E	U	N	E	U	N	E	U
G	non-corr	1.431	1.320	3.450	-	-	-	-	-	-
G/C	non-corr	1.558	2.040	2.955	4.199	6.131	9.651	20.609	14.518	42.372
G/C	tgd-corr	1.347	1.523	2.840	2.775	4.879	6.824	8.059	10.672	17.466
G/C	dcb-corr	1.322	1.282	2.798	2.650	3.230	5.480	5.808	5.470	7.858
G/R	non-corr	1.379	1.289	3.435	-	-	-	-	-	-
G/R/C	non-corr	1.391	1.736	2.955	-	-	-	-	-	-
G/R/C	tgd-corr	1.253	1.352	2.864	-	-	-	-	-	-
G/R/C	dcb-corr	1.196	1.171	2.716	-	-	-	-	-	-
G/C/E	non-corr	1.387	0.992	3.251	3.91	5.823	9.456	20.412	14.312	20.495
G/C/E	tgd-corr	-	-	-	-	-	-	7.821	8.389	14.926
G/C/E	dcb-corr	-	-	-	-	-	-	5.165	5.060	7.361
G/R/C/E	non-corr	0.763	0.768	2.612	-	-	-	-	-	-

**Table 5:** RMS of multi-constellation PPP (unit: m).

Combinations	Scheme	b1b2			b1b3			b2b3		
		N	E	U	N	E	U	N	E	U
G	non-corr	0.008	0.013	0.023	-	-	-	-	-	-
C	non-corr	0.019	0.033	0.056	0.070	0.111	0.182	0.238	0.241	0.456
C	tgd-corr	-	-	-	0.072	0.105	0.182	0.156	0.203	0.421
C	dcb-corr	-	-	-	0.071	0.106	0.166	0.142	0.180	0.398
G/R	non-corr	0.008	0.013	0.024	-	-	-	-	-	-
G/C	non-corr	0.008	0.012	0.022	0.058	0.093	0.139	0.226	0.209	0.433
G/C	tgd-corr	-	-	-	0.046	0.079	0.108	0.141	0.192	0.401
G/C	dcb-corr	-	-	-	0.041	0.046	0.103	0.138	0.172	0.379
G/R/C	non-corr	0.009	0.014	0.024	-	-	-	-	-	-
G/C/E	non-corr	0.012	0.025	0.034	0.041	0.070	0.107	0.203	0.208	0.413
G/C/E	tgd-corr	-	-	-	0.039	0.070	0.097	0.140	0.189	0.391
G/C/E	dcb-corr	-	-		0.037	0.065	0.093	0.135	0.170	0.375
G/R/C/E	non-corr	0.009	0.014	0.025	-	-	-	-	-	-

**Table 6:** RMS of multi-constellation SPP (unit: m).

G/R/C	b1			b2			b1b2		
	N	E	U	N	E	U	N	E	U
non-corr	3.425	2.583	10.220	4.227	2.757	12.794	3.353	3.357	2.725
tgd-corr	2.504	1.309	8.610	3.612	1.755	9.691	1.168	0.936	2.485
dcb-corr	2.009	1.009	8.590	3.760	1.264	9.035	1.213	0.916	2.444
